# Suicide attempt among Malaysian school-going adolescents: relationship with bullying

**DOI:** 10.1186/s12889-023-17019-2

**Published:** 2023-11-06

**Authors:** Siaw Hun Liew, Mohamad Aznuddin Abd Razak, Mohd Shaiful Azlan Kassim, Noor Ani Ahmad, LeeAnn Tan

**Affiliations:** Institute for Public Health, National Institutes of Health, Ministry of Health Malaysia, Shah Alam, Malaysia

**Keywords:** Adolescents, Malaysia, School bullying, Suicide attempt

## Abstract

**Background:**

According to World Health Organization (WHO), the fourth leading cause of death among adolescents aged 15 -19 years is suicide. The National Health and Morbidity Survey (NHMS) 2017 reported that suicidal attempts among school adolescents increased from 6.8%—6.9% as compared to NHMS 2012. Suicide attempts can cause significant negative impacts on health, social and economic status. Bullying is one of the factors for adolescent suicide attempts, and its relationship to suicidality in adolescents has been shown in numerous research.

**Objectives:**

This study examined the relationship between suicide attempts and bullying among school adolescents in Malaysia.

**Methods:**

Data from the Malaysia NHMS 2017, a nationwide study that adopted a two-stage cluster sampling design, were analysed. The survey used a self-administered questionnaire in bilingual language adapted from GSHS developed by WHO. Participants were secondary school students aged 13 -17 in all states. Descriptive and multiple logistic regression analyses were performed using IBM SPSS version 28.

**Results:**

A total of 27,497 school adolescents participated in the study. Results showed that 6.9% of school adolescents had attempted suicide. There was 16.2% of adolescents being bullied. Multiple logistic regression revealed that students who were bullied were more likely to have suicide attempts (aOR 4.827, 95% CI: 4.143, 5.624) *P* < *0.001.*

**Discussion/conclusion:**

This study revealed that bullying is associated with suicide attempts among school adolescents in Malaysia. The respective authority should consider and plan effective measures to curb bullying among school adolescents.

## Introduction

Adolescence is between the ages of 10 and 19 when a person transitions from being a "child" to an "adult." Significant physical, psychological, and behavioral changes occur throughout these formative and impressionable years [1].

One in seven 10 to 19-year-old adolescents worldwide reported having a mental disorder, which accounts for 13% of the overall global burden of disease in this age range [2]. Adolescent suicide has been a public health concern worldwide, as discussed in numerous articles. The fourth leading cause of death among adolescents aged 15 to 19 years is suicide, as reported by the World Health Organization (WHO), 2021 [3]. In Malaysia, compared to National Health and Morbidity Survey (NHMS) 2012 [4], there was an increase in suicidal ideation, suicidal plans, and suicide attempts from 7.9% to 10%, 6.4% to 7.3% and 6.8%—6.9%, respectively as reported by NHMS 2017) [5].

Suicidal behaviors can be classified as suicidal ideation, suicidal plans, and suicide attempts [6]. Suicide attempts are the most serious of the three as they can cause significant negative impacts on health, social and economic status including medical services expenditure in treating injuries and subsequent permanent impairments. Suicide attempts can also have a devastating psychological impact on individuals and family members. Additionally, adolescents who have attempted suicide are at higher risk of substance abuse, engaging in violent behavior, experiencing mental health problems, physical health issues, and premature death in adulthood [7, 8]. Suicide is defined as a fatal self-injurious act with some evidence of intent to die [9]. Suicide is when people themselves in an attempt to end their life. A suicide attempt is when people harm themselves, intending to end their lives, but they survive [10].

Many associated risk factors may contribute to suicidal behaviour among adolescents, including mental disorders such as depression, anxiety; bullying; poor parental support; peer relationships, etc. Additionally, there is a strong relationship between suicidal behavior and violence, abuse, experiencing conflict, disaster, or loss, and a sense of isolation [11].

Youth violence is a global public health problem. It includes a range of acts from bullying and physical fighting to more severe sexual and physical assault to homicide. Bullying can be physical or cyberbullying. A study of 40 developing countries showed that an average of 42% of boys and 37% of girls were exposed to bullying [12]. Between 20 and 56% of young adolescents are involved in bullying annually [13]. Malaysia Global School-based Student Health Survey in 2012 reported 17.9% of school adolescents being bullied [4]. School bullying is the most common type of youth violence found in a Korean study the result showed a 40% prevalence of bullying among middle-school students [14]. The prevalence of cyberbullying victimization is 13.7% among school adolescents in Peninsular Malaysia reported in a recent literature in Malaysia [15].

Centers for Disease Control and Prevention, 2014 defined bullying as unwanted, aggressive behavior among school-aged children that involves a real or perceived power imbalance [16]. Over time, the behavior is repeated and has the potential to be repeated. Bullying includes spreading rumours, threatening, physically or verbally assaulting, and isolating someone from a group purposely. Bullying has detrimental long-term effects on the mental health and general well-being of adolescents. Adolescents involved in bullying, whether they are bullies or victims, have a higher risk for suicide-related behavior.

Among the risk factors for suicide attempts in adolescents, bullying can be a trigger factor that needs to be given attention. There have been several research on bullying and suicide attempts that reported a direct relationship between bullying victimization and adolescent suicides which is a much more serious condition. Being bullied in adolescence is a significant risk factor for self-harm and suicidal behavior [17]. Both traditional bullying and cyberbullying were associated with suicidality among adolescents as found by a study in China [18]. In high-income countries, bullying victimization has been identified as a significant risk factor for adolescent suicidal behaviors [19]. In examining the relationship between bullying victimization and suicidal behaviors among adolescents, Holt et al*.* (2015) found that bullying among adolescents was associated with suicide attempts [20]. A study among middle schoolers in the United States found out bullying victims were more likely to attempt suicide than those who had not been bullied [21]. A meta-analysis research that analysed data from 9 studies, including 1 Low or Middle-income Country (South Africa), and 4 high-income countries (Sweden, United States, New Zealand, and Ireland), found that bullying victims were at a 2.55 times higher risk of suicide attempts [22]. Findings from a multi-country study with 10 European nations revealed that victims of bullying had a 1.26 times higher risk of suicide attempts [23]. According to a Canadian longitudinal study, bullying victimization at the age of 13 increased the risk of suicide attempts 2 years later by 3.05 times [24].

Most studies were done on suicidal behavior and associated factors among adolescents in Malaysia, however, there is no study focused on suicide attempts and bullying among Malaysian School-going adolescents. In Malaysia, school bullying is a pervasive issue that affects students, teachers, and parents. School bullying is still a persistent issue despite increased efforts to overcome it. A total of 120 cases had already been reported by August 2022, highlighting the need for urgent action to address this problem [25]. In order to ensure that the schools are bully-free and safe environments for students, The Malaysian Education Ministry (MOE) and Ministry of Home Affairs launched a bullying complaint channel in the MOE portal in August 2022 for parents/students to report bullying that occurs among students in schools [26].

Thus, this study aims to identify the prevalence of suicide attempts and bullying and the relationship between bully and suicide attempts among Malaysian school-going adolescents using nationally representative data.

## Methodology

### Study design and sampling

Data were obtained and analysed from a nationwide survey in Malaysia, the National Health and Morbidity Survey (NHMS) 2017 [5]. This was a cross-sectional study conducted from 26^th^ March to 3^rd^ May 2017. The survey used a two-stage stratified cluster random sampling method and sampling weights were calculated to ensure representativeness to the general population. A sampling of the selection of secondary schools with students from Form 1 to Form 5 was done in the first stage of the study. Schools were selected randomly with probability proportionate to school enrolment size. From a total of 2,738 schools, 212 secondary schools were selected to participate in this survey. In the second stage of sampling, the selection of classes in each selected school was done by systematic random sampling. All students in selected classes were invited to participate in the survey. Further details on the study’s design and sampling methodology can be found in Awaluddin et al*.* (2019) [27].

### Instrument

The survey used a self-administered questionnaire in bilingual language adapted from the Global School-based Student Health Survey (GSHS) developed by the World Health Organization, which has been utilised globally, translated, and validated in Malaysia [4, 5, 27]. It was used in Malaysia GSHS 2012 [4] with computer-scannable answer sheets. It contains 10 main health risk behaviors among adolescents including suicidal behavior. To ensure the confidentiality of the participants, answer sheets were made anonymous.

### Data collection

**T**he National Health and Morbidity Survey (NHMS) 2017 [5] was approved by both the Ministry of Health and the Ministry of Education of Malaysia. The study obtained ethical approval from the Medical Research and Ethics Committee (MREC) Ministry of Health Malaysia (Approval code: NMRR-16–698-30,042). A consent form was given to each student from selected schools in order to obtain their parents'/guardians' approval seven days before the survey. Before the survey started on the day of the survey, student consent was obtained from those eligible respondents. Students who refused to participate in the survey or did not acquire parental approval were considered as non-responses of eligible participants in the survey.

A brief explanation of the questionnaire survey was given to students who consented to the study; they were assured of the confidentiality of their information, and study data would only be used for the Ministry of Health research purposes. The participants were then given a self-administered questionnaire sheet. Further explanation was provided for any incomprehensible questionnaire items.

### Definition of variables

The NHMS 2017 measures were used as the variables for analysis in this research. Suicidal attempt refers to when the respondents attempted suicide at least once in the past 12 months prior to the survey. Suicide attempts were measured by asking the respondent “During the past 12 months, how many times did you actually attempts suicide?’. There were several response options, including *“0 times”, “1 time”, “2 or 3 times”, “4 to 5 times”, and “6 or more times”.* They were reported as having attempted suicide if they had attempted suicide at least one time in the past 12 months before the survey.

In NHMS 2017 [5], bullying is defined as “when a student or group of students say or do bad and unpleasant things to another student, such as teasing a lot in an unpleasant way or leaving out of things on purpose in the past 30 days. It is NOT bullying when two students of about the same strength or power argue or fight or when teasing is done in a friendly and fun way.” To measure bullying victimization, respondents were asked the following question: “During the past 30 days, on how many days were you bullied?” The options of answers available were *“0 days”, “1 or 2 days”, “3 – 5 days”, “6 – 9 days”, “11 – 19 days”, “20 – 29 days”, and all “30 days*”. Respondents were considered as having been bullied if they experienced bullying on 1 or more days in the last 30 days prior to the survey.

### Data analysis

Statistical Package for Social Science (SPSS) version 28 was used to analyse data. The prevalence of demographic characteristics, suicide attempts, and bullied was generated using a complex sampling design and described in descriptive statistics (Table 1). The univariate analysis determined the association between suicidal attempts and bullied and binary logistic regression. Then, a multiple logistic regression model was conducted to prove the association of suicidal attempts with being bullied. The result was reported in odds ratios (OR), 95% confidence interval (CIs), and *P* value < 0.05. The results were then tabulated as in Table 2. Multi-collinearity and interaction were checked and not found. Model fitness was tested.

**Table 1:** Prevalence of suicidal attempts, bullying, and other socio-demo factors among Malaysian school-going adolescents (*N* = 27,497)

Variables	Categories	n	Estimated Population	%
Gender	Male	13,067	1,058,653	49.6
	Female	14,302	1,075,625	50.4
Age (years)	12 and below	145	12,409	0.6
	13–15	16,807	1,290,090	60.1
	16–17	10,156	814,623	38.0
	18 and older	389	29,324	1.4
Ethnicity	Malay	18,634	1,347,224	63.1
	Chinese	4,083	357,036	16.7
	Indian	1,424	149,064	7.0
	Bumiputera Sabah	1,765	147,460	6.9
	Bumiputera Sarawak	919	96,624	4.5
	Others	544	36,871	1.7
Marital status of parents	Living together	22,527	1,751,332	83.9
	Living apart / divorced/widowed	4,272	335,092	16.1
Bullied at least once	Yes	4,436	346,882	16.2
	No	23,022	1,795,518	83.8
Suicidal attempt	Yes	1,755	148,510	6.9
	No	25,672	1,992,009	93.1

**Table 2:** Multiple logistic regression of bullying associated with suicidal attempts among Malaysian school-going adolescents

Variables	Suicide attempt	
	**AOR (95% C.I)**	***P***
Gender		
Male	0.883 (0.781,0.998)	0.047
Female^a^	1	
Age (years)		
12 and below	1.253 (0.565,2.775)	0.577
13–15	**1.415 (1.151,1.738)**	**0.001**
16-17^a^	1	
18 and above	1.175 (0.565,2.443)	0.665
Ethnicity		
Malay^a^	1	
Chinese	**2.642 (2.197,3.177)**	** < 0.001**
Indian	**3.785 (2.871,4.991)**	** < 0.001**
Bumi Sabah	1.233 (0.888,1.712)	0.210
Bumi Sarawak	**2.086 (1.586,2.743)**	** < 0.001**
Others	**1.965 (1.325,2.915)**	**0.001**
Marital status of parents		
Living together^a^	1	
Living apart	**1.412 (1.223,1.631)**	** < 0.001**
Bullied at least once		
Yes	**4.827 (4.143,5.624)**	** < 0.001**
No^a^	1	

*Abbreviation*: *OR* Odds ratio, *CI* Confidence interval

Multicollinearity and interactions were checked and not found. Nagelkerke 18.4%, classification table (overall correctly classification table = 90.5%) and receiver operating characteristic (ROC) curve (area under ROC curve = 75%) were accepted to check model fitness

Subtotal number of responses differs due to missing data

^a^1 = reference group

^*^—*P* > .25 (binary analysis) not included in multiple logistic regression

## Results

The study included 27,497 school adolescents in total. The overall response rate was 89.2%. In this study, 50.4% of school girls and 49.6% of boys participated as responders. Table 1 displays statistics on the prevalence of suicide attempts among Malaysian school-going students by demographic features and bullying. Findings showed suicide attempts were 6.9% (95%CI: 6.67, 8.05). Overall, 16.2% of school-going students reported experiencing bullying at least once in the past 30 days before the survey.

Multiple logistic regression (Table 2) shows that suicide attempts and bullying are associated. Adolescents who experienced bullying were at a 4 times higher risk of suicide attempts (aOR 4.827, 95% CI: 4.413, 5.624) *P* < *0.001*. According to the findings, adolescents aged 13 – 15 showed a higher risk of suicide attempts among all age groups (aOR 1.415, 95% CI: 1.151,1.738) *P* = *0.001*. Results show that among all ethnicities, Indians were at 3.8 times higher risk of suicide attempts (aOR 3.785, 95% CI: 2.871, 4.991) *P* < *0.001,* followed by Chinese Ethnicity (aOR 2.642, 95% CI: 2.197, 3.177) *P* < *0.001*. Students whose parents live apart show a higher likelihood of suicide attempts (aOR 1.412).

## Discussion

This study examined suicide attempts and the relationship between bullying and suicide attempts, revealing that 6.9% of Malaysian adolescents attempted suicide, and bullying victims had a 4 times higher risk of suicide attempt than non-victims. Our research finding of suicide attempt was higher compared to China (2.9%) [28], Indonesia, Brunei, and Laos where the suicidal attempt was at 3.9%, 5.2%, and 5.9% respectively [29]. However, the result was lower than a survey reported in Thailand whereby 13.3% of Thailand adolescents aged 13 – 17 years old attempted suicide. Our result was also lower compared to Myanmar (8.8%) and Timur-Leste (9.5%) [1]. In addition, a population-based study done by Uddin et al*.* (2019) [30] in 59 low and middle countries involving 29, 129 adolescents showed a higher rate of suicidal attempts compared to our result at 17%. A study by Maniam et al. (2014) [18] revealed that younger people aged 16–24 in Malaysia are at a 2.6 times higher risk of suicidal behavior. According to Wu et al. (2012), compared to other Asian regions, the prevalence of suicidal behaviors among school-going adolescents in Malaysia is comparatively low; non-reporting and underreporting of suicide cases in Malaysia are most probably the reason for the low rate of suicide cases due to cultural, religious as well as legal factors [31]. Additionally, according to the Malaysian Penal Code, Sect. 309, suicide is an offence that can be fined, imprisoned, or both, which further prevents reporting such cases [32]. Suicide is culturally taboo; thus, reporting is avoided to spare families from embarrassment and stigma [31].

This study’s result showed adolescents aged 13 – 14 years reported higher odds of suicide attempts compared with other age groups. The result is not consistent with a past study [33] in which adolescents aged 17 and 18 reported the highest rates at 40.40% and 28.40% respectively. Contrary to another study, a pooled analysis of GSHS data from 90 countries found no difference in suicide attempts by age group in [34].

Results showed gender factors are not significantly related to suicide attempts. This finding is comparable to research done by Campisi et al. (2020) which analysed data from 90 countries of the global school-based student health survey among adolescents [34]. Their result showed no difference in suicide attempt rates for both genders. Our study result is in line with past studies which showed no statistically significant difference between male and female adolescent suicide attempts [35]. However, there were some other studies with contrary findings. Miranda et al. (2019) [36] conducted a meta-analysis of 24 studies and reported that females presented with a two-fold higher risk of suicide attempts than males. A school health survey among adolescents aged 11 -17 years in China reported 8.0% of the respondents attempted suicide, and girls showed a higher rate (9.3%) than boys (6.6%) [37]. More girls (5.34%) reported suicide attempts than boys (2.14) in a study involving adolescents aged 13 – 19 years done in Eastern Poland [33]. Maniam et al*.* (2014) [38] reported that 16% of Malaysian adolescent girls have a higher risk of suicidal behavior. The discrepancy between male and female suicidal behavior rates may be associated with cultural acceptability, psychosocial differences between the two genders, and the suicide method used [39, 40].

The prevalence of suicide attempts differs among ethnicities, where Indians and Chinese were at higher risk of having suicide attempts compared to other ethnicities. Among all ethnicities, Indians were significantly higher in suicide attempts (38%). Other studies in Malaysia have consistently reported higher rates of suicidality among Indians [38, 41–43]. Malaysia is a multiracial society with a diverse population of religious and cultural values. Therefore, the variations in suicide rates among ethnicities are likely influenced by religious and cultural factors.

This study discovered that 16.2% of respondents reported that they had been bullied. This result was lower compared to other South East Asia Countries, whereby the rate of bullying among school adolescents reported by the Philippines (51.5%), Thailand (32.7%), Timor-Leste (31.3%), Brunei (23%), Cambodia (22.2%), and Indonesia (21%), but higher compared with Laos (13.2%) [29]. Our result is lower than the findings reported by Han et al. (2018) where 25.39% of adolescents were bullied traditionally while 8.30% experienced cyberbullying [18]. Our result is higher compared with Korea where 14% of their school adolescents were victims of bullying [14]. This study showed a higher result of bullying compared to findings reported by a study in China where 11.7% of students experienced bullying [44]. The Global School-based Student Health Survey (2009 -2015) from 48 countries reported that the prevalence of bullying victimisation among adolescents aged 12 to 15 was 30.4% [29]. Similarly, the result was shown in a meta-analysis of 80 studies by Modecki et al*.* (2014) that reported a prevalence of bullying victimisation of 36% [45].

Being bullied can be a significant traumatic event associated with many negative effects [46, 47]. The current study has determined a relationship between suicide attempts and bullying, showing that 17% of adolescents who experienced bullying have attempted suicide. This is in line with other works of literature. A study done in China found that for students who were bullied, girls reported 2.6 higher odds of suicide attempts, and boys showed 2 times the odds for suicide attempts [48]. Other South Asian countries such as Bangladesh and Nepal, reported a strong relationship between bullying victimization and suicide attempts of 3 times the risk of attempting suicide among adolescents [49]. Adolescents who experienced traditional bullied were at 1.81 times the risk of suicide attempts, and those victims of cyberbullying were at 2.70 higher odds of suicide attempts [18]. In the Ghana Global School-Based Health Survey, the findings showed 2 times higher odds for suicide attempts among students who were victims of bullying than those who had not experienced bullying [20]. A study done in the United States revealed that traditional bullying victims and cyberbullying victims were 1.7 times and 1.9 times more likely to attempt suicide than adolescents who were not bullying victims respectively [21].

This study's results are consistent with other studies that reported a positive relationship between bullying and suicide attempts among adolescents including the GSHS between 2009 and 2015 found that among adolescents from 48 countries, being bullied at least once in the past 30 days was associated with threefold higher odds for an overall suicide attempt [29]. A study done in Malawi found that 13.4% of school-going adolescents had attempted one or more suicide attempts after being bullied [35]. Romo et al. reported that in Latin America, adolescents who experienced bullying in the past 30 days were three times the odds of suicide attempts [50]. A school health survey in Tanzania found out adolescents who were bullied were about 4 times odds likely for suicide attempts than those not experiencing bullying [51]. This study’s findings are also consistent with other studies that reported adolescents being bullied were significantly more likely to have suicide attempts [16, 52–56].

This study result showed adolescents whose parents live apart showed a higher risk for suicide attempts compared with adolescents residing with both parents (aOR: 1.412). A study done by Garnefski and Diekstra [57] revealed that adolescents from intact families had the lowest likelihood of suicide attempts. According to a child health study in Ontario, adolescents whose parents lived apart or without biological parents had higher rates of suicide attempts [58]. Adolescents who were being raised in an incomplete family structure reported a higher risk for suicide attempts (27.71%, X^2^ 66.73) [33].

### Strength and limitations

The strength of this study is** t**he analysis used in this study, which was based on a substantial, nationally representative sample, enabled a trustworthy extrapolation to Malaysian adolescents in a related age range. In addition, an anonymous version of the GSHS was employed in this study, which is favourable for cross-national comparison. This poll employed a validated bilingual (English and Bahasa Malaysia) questionnaire that is common with students. However, some limitations of the current study include not identifying the causal relationship between bullying and suicidal attempts but only demonstrating the associations. Second, since it was self-reporting, there might be bias in recalling the bullying experience and answering the questionnaire influenced by respondents’ cultural factors.

## Conclusion

The current study showed in Malaysia, suicide attempts among school-going adolescents are associated with bully victimization. The more often someone is bullied, the more overwhelming for him/her. According to a previous study using the strain theory, conflicting pressure in a person’s life would lead to suicide [59]. Experiences of being bullied can affect adolescents’ mental health and result in suicide attempts. Anti-bullying laws decrease bullying incidents [60], therefore this study provides valuable information to education stakeholders and policy-makers regarding the burden of bullying among in-school adolescents. Despite other factors that contribute to suicidal attempts, stakeholders in education and health should think about expanding their focus to include developing effective and evidence-based measures to address bullying among school-going adolescents to prevent suicide attempt before it starts, rather than only focusing on helping those who are already engaging in these behaviors.

## Data Availability

For data protection purposes, the data used for this study are available from the Institute for Public Health, Ministry of Health Malaysia, but access to this data is restricted because they are not publicly available. However, data are available from the author (liewsh@moh.gov.my) upon reasonable request and with permission from the Director General of Health Malaysia.

## References

[1] Mental health status of adolescents in South-East Asia: Evidence for action. New Delhi: World Health Organization, Regional Office for South-East Asia; 2017. Licence: CC BY-NC-SA 3.0 IGO.

[2] World Health Organization. Adolescent mental health. 2021. Cited 2022 Jan 25. Available from: https://www.who.int/news-room/fact-sheets/detail/adolescent-mental-health.

[3] World Health Organization. Suicide. 2021. Cited 2023 Feb 3. Available from: https://www.who.int/news-room/fact-sheets/detail/suicide.

[4] Institute for Public Health (IPH) (2012). The National Health and Morbidity Survey: Malaysia Global School-based Student Health Survey 2012.

[5] Institute for Public Health (IPH) 2017. National Health and Morbidity Survey (NHMS) 2017. Malaysia: Adolescent Health Survey; 2017.

[6] WHO., Saxena S, Saxena S, Krug EG, Krug EG, Chestnov O, et al. Preventing Suicide : a Global Imperative. World Health Organization; 2014. p. 92. Available at https://www.who.int/publications/i/item/9789241564779.

[7] Mars B, Heron J, Crane C, Hawton K, Lewis G, Macleod J (2014). Clinical and social outcomes of adolescent self harm: Population based birth cohort study. BMJ (Online).

[8] Goldman-Mellor SJ, Caspi A, Harrington HL, Hogan S, Nada-Raja S, Poulton R (2014). Suicide attempt in young people a signal for long-term health care and social needs. JAMA Psychiat.

[9] Gvion Y, Apter A (2012). Suicide and suicidal behavior. Public Health Rev..

[10] Institute of Mental Health N. Frequently Asked Questions About Suicide. Available from: https://suicidepreventionlifeline.org.

[11] World Health Organization. Suicide. 2021 Cited 2023 Feb 3. Available from: https://www.who.int/news-room/fact-sheets/detail/suicide.

[12] WHO. Youth Violence. 2020. Cited 2023 Feb 3. Available from: https://www.who.int/news-room/fact-sheets/detail/youth-violence.

[13] Borowsky IW, Taliaferro LA, McMorris BJ (2013). Suicidal thinking and behavior among youth involved in verbal and social bullying: Risk and protective factors. J Adolesc Health..

[14] Kim YS, Koh YJ, Leventhal BL (2004). Prevalence of School Bullying in Korean Middle School Students. Arch Pediatr Adolesc Med..

[15] Fadhli SAM, Yan JLS, Halim ASA, Razak AA, Rahman AA (2022). Finding the Link between Cyberbullying and Suicidal Behaviour among Adolescents in Peninsular Malaysia. Healthcare (Switzerland)..

[16] Centers for Disease Control and Prevention (CDC). The Relationship Between Bullying and Suicide: What We Know and What it Means for Schools; 2014. Available from: https://www.cdc.gov/violenceprevention/pdf/yv/bullying-suicide-translation-final-a.pdf.

[17] Sigurdson JF, Undheim AM, Wallander JL, Lydersen S, Sund AM (2018). The Longitudinal association of being bullied and gender with suicide ideations, self-harm, and suicide attempts from adolescence to young adulthood: a cohort study. Suicide Life Threat Behav.

[18] Han Z, Fu M, Liu C, Guo J (2018). Bullying and suicidality in Urban Chinese youth: the role of teacher-student relationships. Cyberpsychol Behav Soc Netw.

[19] Reed KP, Nugent W, Cooper RL (2015). Testing a path model of relationships between gender, age, and bullying victimization and violent behavior, substance abuse, depression, suicidal ideation, and suicide attempts in adolescents. Child Youth Serv Rev.

[20] Baiden P, Kuuire VZ, Shrestha N, Tonui BC, Dako-Gyeke M, Peters KK (2019). Bullying victimization as a predictor of suicidal ideation and suicide attempt among senior high school students in Ghana: results from the 2012 Ghana Global School-Based Health Survey. J Sch Viol.

[21] Hinduja S, Patchin JW (2010). Bullying, cyberbullying, and suicide. Arch Suicide Res.

[22] Van Geel M, Vedder P, Tanilon J (2014). Relationship between peer victimization, cyberbullying, and suicide in children and adolescents ameta-analysis. JAMA Pediatr.

[23] Barzilay S, Brunstein Klomek A, Apter A, Carli V, Wasserman C, Hadlaczky G (2017). Bullying victimization and suicide ideation and behavior among adolescents in europe: a 10-country study. J Adolesc Health.

[24] Geoffroy MC, Boivin M, Arseneault L, Turecki G, Vitaro F, Brendgen M (2016). Associations between peer victimization and suicidal ideation and suicide attempt during adolescence: results from a prospective population-based birth cohort. J Am Acad Child Adolesc Psychiatry.

[25] Education, Home Ministries launch channel for students and parents to report bullying in schools. https://www.malaymail.com/news/malaysia/2022/08/18/education-home-ministries-launch-channels-for-students-and-parents-to-report-bullying-in-schools/23396. 2022 Aug 18;

[26] Ministry of Education, Malaysia. Portal Aduan Buli. Cited 2023 Oct 16. Available from: https://www.moe.gov.my/aduanbuli.

[27] Awaluddin SM, Ibrahim Wong N, Rodzlan Hasani WS (2019). Methodology and Representativeness of the Adolescent Health Survey 2017 in Malaysia. Asia Pac J Public Health..

[28] Wang H, Bragg F, Guan Y, Zhong J, Li N, Yu M (2023). Association of bullying victimization with suicidal ideation and suicide attempt among school students: A school-based study in Zhejiang Province China. J Affect Disord.

[29] Koyanagi A, Oh H, Carvalho AF, Smith L, Haro JM, Vancampfort D (2019). Bullying victimization and suicide attempt among adolescents aged 12–15 years from 48 countries. J Am Acad Child Adolesc Psychiatry.

[30] Uddin R, Burton NW, Maple M, Khan SR, Khan A (2019). Suicidal ideation, suicide planning, and suicide attempts among adolescents in 59 low-income and middle-income countries: a population-based study. Lancet Child Adolesc Health.

[31] Wu KCC, Chen YY, Yip PSF (2012). Suicide methods in Asia: Implications in suicide prevention. Int J Environl Res Public Health..

[32] Attorney General’s Chambers of Malaysia. Laws of Malaysia, Act 574 - Penal Code. 2018. Cited 2023 Apr 5. 178–179. Available from: https://lom.agc.gov.my/ilims/upload/portal/akta/LOM/EN/Penal%20Code%20ACT%20574%20-%20TP%20LULUS%2021_2_2018.pdf.

[33] Zygo M, Pawłowska B, Potembska E, Dreher P, Kapka-Skrzypczak L (2019). Prevalence and selected risk factors of suicidal ideation, suicidal tendenciesand suicide attempts in young people aged 13–19 years. Ann Agric Environ Med.

[34] Campisi SC, Carducci B, Akseer N, Zasowski C, Szatmari P, Bhutta ZA (2020). Suicidal behaviours among adolescents from 90 countries: A pooled analysis of the global school-based student health survey.

[35] Shaikh MA, Lloyd J, Acquah E, Celedonia KL, Wilson ML (2016). Suicide attempts and behavioral correlates among a nationally representative sample of school-attending adolescents in the Republic of Malawi. BMC Public Health..

[36] Miranda-Mendizabal A, Castellví P, Parés-Badell O, Alayo I, Almenara J, Alonso I (2019). Gender differences in suicidal behavior in adolescents and young adults: systematic review and meta-analysis of longitudinal studies. Int J Public Health..

[37] Cui S, Cheng Y, Xu Z, Chen D, Wang Y (2011). Peer relationships and suicide ideation and attempts among Chinese adolescents. Child Care Health Dev.

[38] Maniam T, Marhani M, Firdaus M, Kadir AB, Mazni MJ, Azizul A (2014). Risk factors for suicidal ideation, plans and attempts in Malaysia - Results of an epidemiological survey. Compr Psychiatry..

[39] Bilsker D, White J (2011). The silent epidemic of male suicide. BC Med J.

[40] Beautrais AL (2003). Suicide and serious suicide attempts in youth: a multiple-group comparison study. Am J Psychiatry.

[41] Ahmad NA, Cheong SM, Ibrahim N, Rosman A (2014). Suicidal ideation among Malaysian adolescents. Asia-Pacific journal of public health / Asia-Pacific Academic Consortium for Public Health..

[42] Chen PCY, Lai KL, Kam CW, Kaur J (2005). Factors relating to adolescent suicidal behavior: a cross-sectional Malaysian school survey. J Adolesc Health.

[43] Morris P, Maniam T (2001). Ethnicity and Suicidal Behaviour in Malaysia: A Review of the Literature. Transcult Psychiatry.

[44] Zhang YY, Lei YT, Song Y, Lu RR, Duan JL, Prochaska JJ (2019). Gender differences in suicidal ideation and health-risk behaviors among high school students in Beijing, China. J Glob Health.

[45] Modecki KL, Minchin J, Harbaugh AG, Guerra NG, Runions KC (2014). Bullying prevalence across contexts: A meta-analysis measuring cyber and traditional bullying. J Adolesc Health..

[46] Bannink R, Broeren S, Van De Looij - Jansen PM, De Waart FG, Raat H. Cyber and traditional bullying victimization as a risk factor for mental health problems and suicidal ideation in adolescents. PLoS One. 2014;9(4):1–7. 10.1371/journal.pone.0094026. 10.1371/journal.pone.0094026 PMC3981739 24718563

[47] Sampasa-Kanyinga H, Roumeliotis P, Xu H (2014). Associations between cyberbullying and school bullying victimization and suicidal ideation, plans and attempts among Canadian schoolchildren. PLoS One..

[48] Yang T, Guo L, Hong F, Wang Z, Yu Y, Lu C (2020). Association between bullying and suicidal behavior among chinese adolescents: An analysis of gender differences. Psychol Res Behav Manag.

[49] Rahman MM, Rahman MM, Khan MMA, Hasan M, Choudhury KN (2020). Bullying victimization and adverse health behaviors among school-going adolescents in South Asia: Findings from the global school-based student health survey. Depress Anxiety.

[50] Romo ML, Kelvin EA. Impact of bullying victimization on suicide and negative health behaviors among adolescents in Latin America. Rev Panam Salud Publica. 2016;40(5):347–55. Available from: http://www.cdc.gov/gshs/. 28076584

[51] Shayo FK, Lawala PS (2019). Does bullying predict suicidal behaviors among in-school adolescents? A cross-sectional finding from Tanzania as an example of a low-income country. BMC Psychiatry..

[52] Arango A, Opperman KJ, Gipson PY, King CA (2016). Suicidal ideation and suicide attempts among youth who report bully victimization, bully perpetration and/or low social connectedness. J Adolesc.

[53] Copeland WE, Wolke D, Angold A, Costello EJ (2013). Adult psychiatric outcomes of bullying and being bullied by peers in childhood and adolescence. JAMA Psychiat.

[54] Espelage DL, Holt MK (2013). Suicidal ideation and school bullying experiences after controlling for depression and delinquency. J Adolesc Health..

[55] Hertz MF, Donato I, Wright J. Bullying and suicide: a public health approach. J Adolesc Health. 2013;53:S1–3. USA: Elsevier. 10.1016/j.jadohealth.2013.05.002 PMC4721504 23790194

[56] Hepburn L, Azrael D, Molnar B, Miller M (2012). Bullying and suicidal behaviors among urban high school youth. J Adolesc Health.

[57] Garnefski N, René R, Diekstra FW. Adolescents from one parent, stepparent and intact families: emotional problems and suicide attempts. J Adolesc. 1997;20:201–08. 10.1006/jado.1996.0077 9104655

[58] Georgiades K, Boylan K, Duncan L, Wang L, Colman I, Afifi TO (2019). Prevalence and Correlates of Youth Suicidal Ideation and Attempts: Evidence from the 2014 Ontario Child Health Study. Can J Psychiat.

[59] Zhang J, Dong N, Delprino R, Zhou L (2009). Psychological strains found from in-depth interviews with 105 Chinese rural youth suicides. Arch Suicide Res.

[60] Rees DI, Sabia JJ, Kumpas G (2022). Anti-bullying laws and suicidal behaviors among teenagers. J Policy Anal Manage.

